# Complications of thread lift about skin dimpling and thread extrusion

**DOI:** 10.1111/dth.13446

**Published:** 2020-05-19

**Authors:** Cheng‐Kun Wang

**Affiliations:** ^1^ College of Management Chang Jung Christian University Tainan City Taiwan; ^2^ College of Medicine Chung Hwa University of Medical Technology Tainan City Taiwan; ^3^ Department of Dermatology E‐Champ Dermatology Clinic Tainan City Taiwan; ^4^ National Cheng Kung University Hospital National Cheng Kung University Tainan City Taiwan

Dear Editor,

Thread lift is known to be a minimally invasive procedure for facial rejuvenation. Barbed absorbable threads become the mainstream of thread lift, including cog threads and cone threads. The efficacy of thread lift depends on three factors: fixation point, vector, and exit point. The main target layer of threads is subcutaneous layer. How to design thread numbers, positions, depth, and vector is an art. We report three patients with complications of thread lift about dimpling and thread extrusion. We treated skin dimpling with manual therapy and subcision, and removed thread when thread extrusion occurred.

The satisfaction level of thread lift from patients and surgeons increased over time. Recent research results show that thread lift for facial rejuvenation was safe, effective, and has fewer complications.^1^ Initial use of nonabsorbable threads produced side effects and had a frequent complication and revision rate. Newer published methods using absorbable sutures have shown safer and more effective.^2^ The complications of silhouette thread included temporal pain, visible dermal pinching, hematoma, asymmetry, and suture palpability.^3^ The complications of polydioxanone (PDO) thread included dimpling, bruise, asymmetry, thread extrusion, and malar eminence accentuation.^4^

- Case 1: A 29‐year‐old woman presented to our department with thread extrusion on left face for 1 week after she went abroad for thread lift with unknown threads. We found that these barbed threads was too thick to be absorbed. We removed the threads in the direction opposite that of placement (Figures 1 and 2).
- Case 2: A 40‐year‐old woman accepted barbed thread lift and had skin dimpling. These depressions were caused by the cogs of threads. The cogs were not strong. Manual therapy was applied on her face to release the tension of cog threads in the direction opposite that of placement (Figures 3 and 4).
- Case 3: A 39‐year‐old woman accepted barbed thread lift. Anchoring method of the exit point of thread was applied to her face and resulted in the severe skin dimpling on her left face. The dimpling was due to dermis hooked by thread anchor and manual therapy was in vain. We treated the skin dimpling by 18‐G needle subcision (Figure 5). After three times therapies in 3 months, the threaded areas returned to normal skin appearance.

**FIGURE 1:**
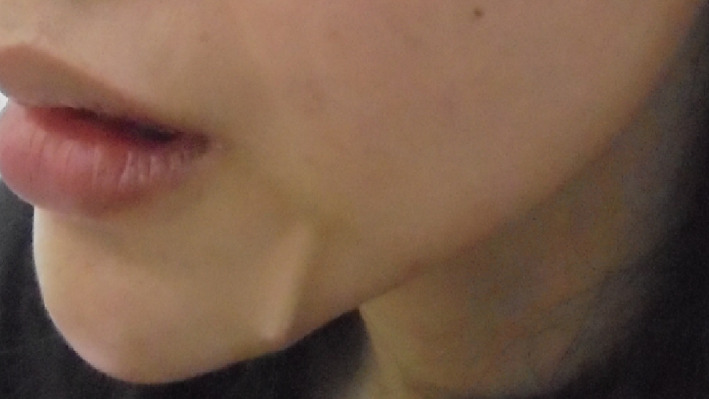
Thread extrusion on left face

**FIGURE 2:**
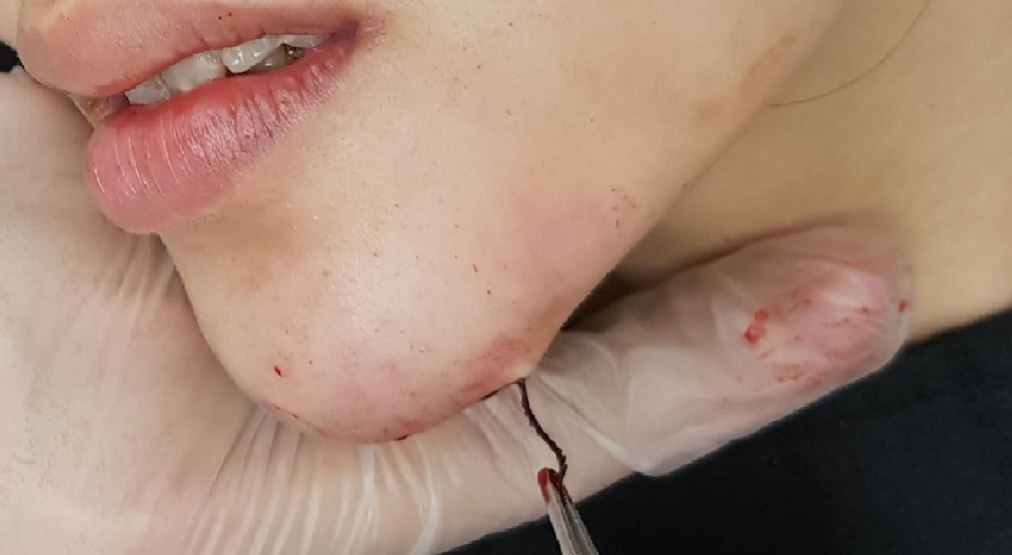
Treat thread extrusion by removing the thread

**FIGURE 3:**
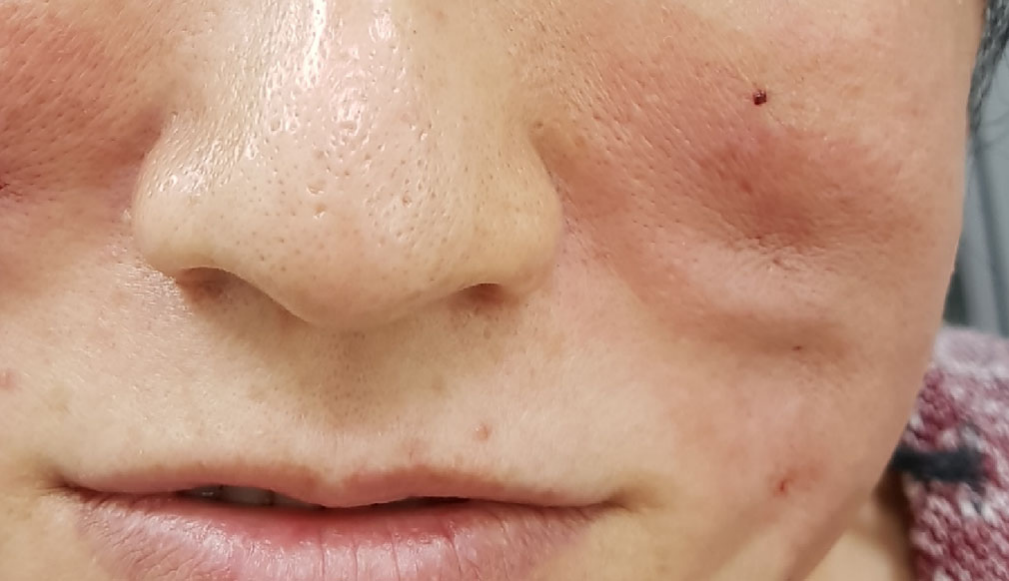
Skin dimpling after thread lift

**FIGURE 4:**
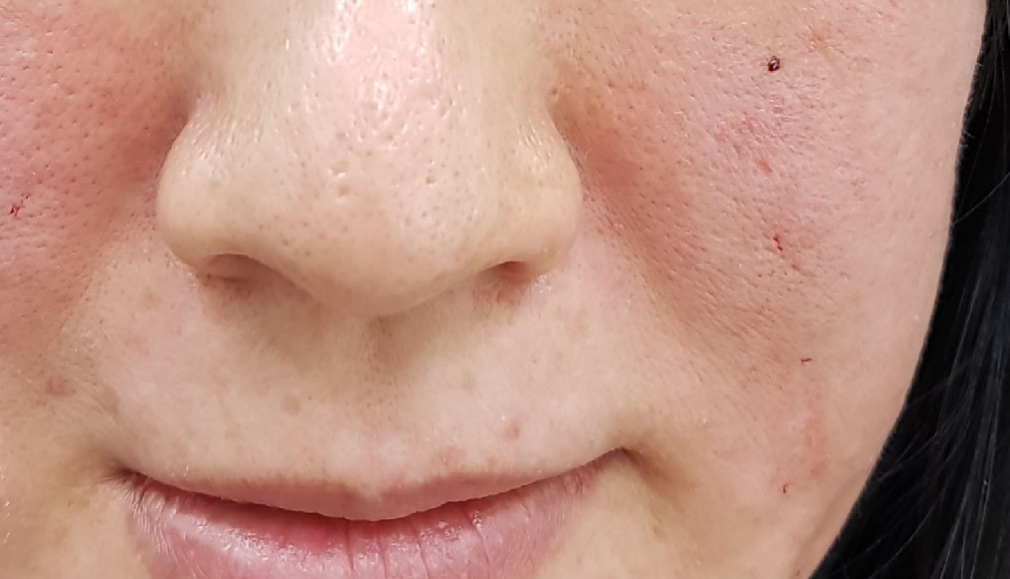
Manual therapy release the tension of cog threads in the direction opposite that of placement

**FIGURE 5:**
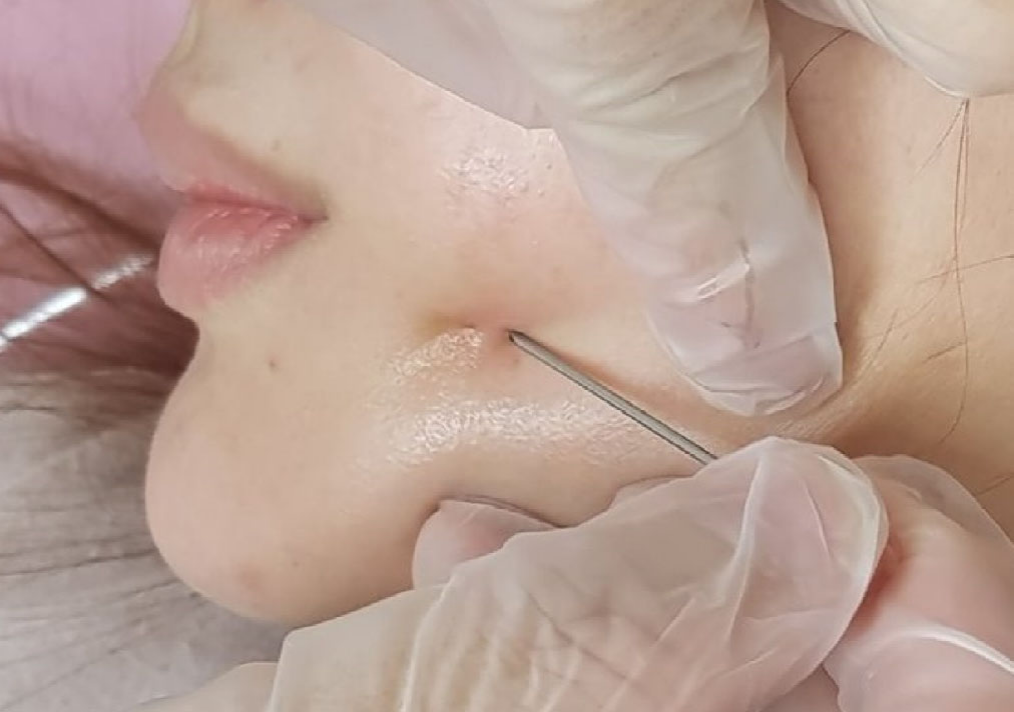
Treat the skin dimpling by 18‐G needle subcision

Treatments of complications of thread lift are highly valued. Suture removal is necessary when displacement of the barbed threads happen. Bertossi et al advised the patient to massage the thread three times a day for 6 days, and the threads subsequently were extracted by the surgeon in the direction opposite that of placement. Mild skin dimpling, erythema, infection, and temporary facial stiffness that happened occasionally.^5^

Lee et al published a study about PDO thread for Asians. The complications included swelling, bruising, skin dimpling, and asymmetry.^6^ Sarigul Guduk and Karaca published a study about 148 patients. The most common complication was skin dimpling and irregularity. The other complications were ecchymosis, suture extrusion, pain, hematoma, infection, and suture migration.^7^ Guida et al published a study about poly‐l‐lactic acid traction thread treatment for mild‐moderate degree of skin laxity of jawline and neck angle. The complications included edema, swelling, temporary skin contour irregularities, and paresthesia.^8^ Archer and Garcia published a study about silhouette instalift thread. The complications includes minor pain, swelling, rippling, dimpling, bruising, bleeding at the entry or exit sites, edema, asymmetry, dysesthesia, skin irregularities, and inflammatory reactions.^9^

In these three cases, Case 3 had the severe dimpling complication. The anchor of the thread hooked the dermis too tight. We used 18‐G needle to cut the thread in the dermis. During subcision, a needle cut these thread thereby releasing the tension of the dermis, allowing the skin to rise. Other than dimpling, prevention of severe complications is very important. Doctors should avoid injuring the superficial temporal artery, zygomatico‐orbital artery, transverse facial artery, facial nerve, and parotid duct.

## CONFLICT OF INTEREST

The authors declare no potential conflict of interest.

## References

[1] Rezaee Khiabanloo S , Jebreili R , Aalipour E , Saljoughi N , Shahidi A . Outcomes in thread lift for face and neck: a study performed with silhouette soft and promo happy lift double needle, innovative and classic techniques. J Cosmet Dermatol. 2019;18:84‐93. 30105878 10.1111/jocd.12745

[2] Tong LX , Rieder EA . Thread‐lifts: a double‐edged suture? A comprehensive review of the literature. Dermatol Surg. 2019;45:931‐940. 30893160 10.1097/DSS.0000000000001921

[3] de Benito J , Pizzamiglio R , Theodorou D , Arvas L . Facial rejuvenation and improvement of malar projection using sutures with absorbable cones: surgical technique and case series. Aesthetic Plast Surg. 2011;35:248‐253. 20835823 10.1007/s00266-010-9570-2

[4] Kang SH , Byun EJ , Kim HS . Vertical lifting: a new optimal thread lifting technique for asians. Dermatol Surg. 2017;43:1263‐1270. 28430736 10.1097/DSS.0000000000001169

[5] Bertossi D , Botti G , Gualdi A , et al. Effectiveness, longevity, and complications of facelift by barbed suture insertion. Aesthet Surg J. 2019;39:241‐247. 29474522 10.1093/asj/sjy042

[6] Lee H , Yoon K , Lee M . Outcome of facial rejuvenation with polydioxanone thread for Asians. J Cosmet Laser Ther. 2018;20:189‐192. 29271683 10.1080/14764172.2017.1400167

[7] Sarigul Guduk S , Karaca N . Safety and complications of absorbable threads made of poly‐L‐lactic acid and poly lactide/glycolide: experience with 148 consecutive patients. J Cosmet Dermatol. 2018;17:1189‐1193. 29607627 10.1111/jocd.12519

[8] Guida S , Persechino F , Rubino G , Pellacani G , Farnetani F , Urtis GG . Improving mandibular contour: a pilot study for indication of PPLA traction thread use. J Cosmet Laser Ther. 2018;20:465‐469. 29461124 10.1080/14764172.2018.1427875

[9] Archer KA , Garcia RE . Silhouette instalift: benefits to a facial plastic surgery practice. Facial Plast Surg Clin North Am. 2019;27:341‐353. 31280848 10.1016/j.fsc.2019.03.006

